# RGS10 mitigates high glucose-induced microglial inflammation via the reactive oxidative stress pathway and enhances synuclein clearance in microglia

**DOI:** 10.3389/fncel.2024.1374298

**Published:** 2024-05-15

**Authors:** Jaegwon Chung, Janna Jernigan, Kelly B. Menees, Jae-Kyung Lee

**Affiliations:** Department of Physiology and Pharmacology, University of Georgia College of Veterinary Medicine, Athens, GA, United States

**Keywords:** microglia, oxidative stress, alpha-synuclein, phagocytosis, neuroinflammation

## Abstract

Microglia play a critical role in maintaining brain homeostasis but become dysregulated in neurodegenerative diseases. Regulator of G-protein Signaling 10 (RGS10), one of the most abundant homeostasis proteins in microglia, decreases with aging and functions as a negative regulator of microglia activation. RGS10-deficient mice exhibit impaired glucose tolerance, and high-fat diet induces insulin resistance in these mice. In this study, we investigated whether RGS10 modulates microglia activation in response to hyperglycemic conditions, complementing our previous findings of its role in inflammatory stimuli. In RGS10 knockdown (KD) BV2 cells, TNF production increased significantly in response to high glucose, particularly under proinflammatory conditions. Additionally, glucose uptake and GLUT1 mRNA levels were significantly elevated in RGS10 KD BV2 cells. These cells produced higher ROS and displayed reduced sensitivity to the antioxidant N-Acetyl Cysteine (NAC) when exposed to high glucose. Notably, both BV2 cells and primary microglia that lack RGS10 exhibited impaired uptake of alpha-synuclein aggregates. These findings suggest that RGS10 acts as a negative regulator of microglia activation not only in response to inflammation but also under hyperglycemic conditions.

## Introduction

Although etiologies of neurodegenerative diseases including Alzheimer’s diseases (AD) and Parkinson’s disease (PD) are not clearly identified, epidemiological studies indicate that they are accompanied by a slew of dysregulated cellular and molecular mechanisms of which misfolded protein aggregates, glucose metabolism, and chronic inflammation appear particularly important (Hoyer, 2004; Chen et al., 2016). Glucose metabolism is responsible for many integral cellular processes in the central nervous system (CNS) such as the production of acetyl CoA, the precursor for the neurotransmitter acetylcholine, and the production of ATP and ATP dependent processes such as those involved in promotion of proper protein synthesis, maintenance, transport and degradation, and maintenance of synaptic transmission (Hoyer, 2000). Hyperglycemia, a condition described by excess glucose in the blood stream, can cause brain damage by increasing oxidative stress and advanced glycation end products (AGEs) which are thought the be an important factor behind neurological conditions associated with diabetes and AD (Sima, 2003). Importantly, it has been demonstrated that up to 80% of AD patients have either impaired glucose tolerance or full-blown diabetes, with diabetes alone recognized as a risk factor for developing dementias (Janson et al., 2004; Biessels et al., 2006; Li et al., 2015).

Microglia are the phagocytic resident immune cells of the CNS and are involved in development, surveillance, maintenance, defense, and inflammatory response of the CNS (Graeber and Streit, 2010). In response to abnormal stimulation such as infections, injury, cellular debris, and abnormal protein aggregations, microglia become activated and undergo morphologic and phenotypic changes (Dheen et al., 2007). When microglia are activated, they produce proinflammatory cytokines, chemokines and reactive oxygen species (Block and Hong, 2005). Microglial activation plays a protective role; however, when microglial activation is sustained, excessive levels of proinflammatory cytokines, chemokines and reactive oxygen species can damage healthy neurons. This further activates microglia, resulting in a deleterious feedback loop that contributes to neurodegeneration (Lull and Block, 2010). Microglia predominantly rely on the glucose transporter 1 (GLUT1) for facilitating glucose uptake, especially during inflammatory conditions (Wang et al., 2019) and hexokinase (HK) 2 was shown to be induced in hypoxia-activated microglial cells (Li et al., 2018). Silencing of GLUT-1 and HK2 genes abolished LPS-induced microglial cell activation (Cheng et al., 2021).

The regulator of G-protein signaling (RGS) protein family is the GTPase-accelerating proteins (GAPs) for specific Gα subunits that negatively regulate G-protein coupled receptor (GPCR) signaling (Guan and Han, 1999). RGS10 is one of the smallest members of the RGS family proteins and is mainly expressed in the brain, bone marrow, testis, and lymph nodes (Hunt et al., 1996; Gold et al., 1997). Unlike other RGS proteins, RGS10 is mainly found in the cytoplasm and translocates to the nucleus upon phosphorylation mediated by cAMP-dependent kinase A (PKA) (Burgon et al., 2001) implicating its role in cellular functions besides modulating GPCR signaling. The level of RGS10 is significantly enriched in microglia (Butovsky et al., 2014) and downregulated with aging and neurodegenerative diseases (Kannarkat et al., 2015; Krasemann et al., 2017). Previously, we have demonstrated RGS10 protein negatively regulates microglia activation by inhibiting the NF-κB pathway and therefore attenuating inflammatory responses (Lee et al., 2008, 2011). Recently, we characterized a new phenotype of RGS10 knockout mice in which RGS10 deficient mice display impaired glucose tolerance and when fed a high-fat diet (HDF). RGS10 deficient mice also exhibit insulin resistance and systemic inflammation (Fang et al., 2019). However, the manner in which RGS10 deficient microglia respond to glucose has yet to be elucidated. BV2 microglia cells exert similar phenotypes of primary microglia (Henn et al., 2009), and we have demonstrated that the level of RGS10 protein in BV2 microglia cells is modulated similarly in response to proinflammatory stimuli as in primary microglia (Lee et al., 2008). Importantly, the loss of RGS10 in BV2 microglia cells enhances proinflammatory phenotypes in response to proinflammatory stimuli (Lee et al., 2008, 2011; Alqinyah et al., 2017, 2018). In this study, we investigated the ability of RGS10 to modulate microglia activation in response to high glucose. Furthermore, high glucose augments proinflammatory cytokine expression in BV2 microglia (Quan et al., 2011; Zhang et al., 2015). We also examined how it changes phagocytic activity of microglia against amyloid alpha-synuclein by utilizing BV2 microglial cells with and without RGS10.

## Materials and methods

### Cell culture

The murine microglia cell line BV2 was cultured in Dulbecco’s modified Eagle’s medium nutrient mixture F-12 (DMEM/F-12) medium supplemented with 10% heat-inactivated fetal bovine serum and 1% penicillin/streptomycin at 37°C in a 5% CO_2_ atmosphere. RGS10 knockdown (KD) BV2 cell line that have previously established and published (Alqinyah et al., 2018; Wendimu et al., 2021) was kindly gifted from Dr. Shelley Hooks at University of Georgia.

Primary microglial cells were obtained from mouse pups (*n* = 6–8 per genotype) aged between postnatal day 3 and 6 (P3–P6), following the procedure described previously (Lee et al., 2011). To summarize, the brain cortices were isolated and finely chopped, then dissociated in 0.25% Trypsin–EDTA for 20 min at 37°C with agitation every 5 min. Subsequently, the trypsin was neutralized using complete medium [DMEM/F12 supplemented with 20% heat-inactivated fetal bovine serum (Sigma), 1% penicillin/streptomycin, and 1% L-glutamine (Sigma)]. The mixed glial cultures were then maintained in complete medium at 37°C with 5% CO2 for 14–18 days *in vitro*. Upon reaching 95% confluence, primary microglial cells were harvested via mechanical agitation (150 rpm for 40 min). The isolated microglia were plated in DMEM/F12 supplemented with 10% heat-inactivated fetal bovine serum. The purity of the microglial cultures was confirmed to be 95% through CD68 (macrosialin) staining, with contamination from astrocytes (GFAP-positive cells) and neurons (MAP2-positive cells) being less than 5%.

### TNF ELISA

TNF alpha Mouse Uncoated ELISA Kit (Invitrogen) was utilized to measure TNF cytokine according to manufacturer’s protocol. Briefly, BV2 cells (2 × 10^4^/well) were plated in a 96 well plate and treated with various reagents. After treatment, supernatants were collected. 96-well plates were incubated with 100 μL of capture antibody per well overnight in the 4°C. The plate was then washed and blocked with 200 μL of 1 × Diluent for 2 h. The plate were washed and incubated with 100 μL of standard and samples for 1 h, followed by incubation with 100 μL of detection antibody for 1 h. TNF levels were detected by incubating with 100 μL of Avidin-HRP for 30 min. Plates were then washed and incubated with 100 μL of 1 × 3,3′,5,5’-Tetramethylbenzidine (TMB) solution for 15 min. The plate was read at 450 nm.

### Measurement of reactive oxygen species

To measure ROS production, we used the oxidation of 2′,7’dichlorofluorescin-diacetate (DCFDA) and measured fluorescent compound 2′,7’dichlorofluorescin (DCF) in the presence of ROS. Briefly, BV2 cells (3 × 10^4^ cells/well) were plated in 96 well plates and treated with various reagents. 10 μM of DCFDA was added into each well and plate was read at excitation and emission wavelengths of 500 nm and 529 nm.

### Cell viability assay

Cell viability was measured by using the CellTiter 96 AQ_ueous_ Assay reagent (Promega, Madison, WI, United States) which utilizes the novel tetrazolium compound (3-(4,5-dimethylthiazol-2-yl)-5-(3-carboxymethoxyphenyl)-2-(4-sulfophenyl)-2H-tetrazolium, inner salt; MTS) and the electron coupling reagent, phenazine methosulfate (PMS). BV2 cells (1 × 10^4^/well) were plated in 96 well plates and treated with low glucose (LG) (17.5 mM), high glucose (HG) (35 mM), or mannitol (35 mM) or LPS (10 ng/mL). MTS/PMS solutions were added into each well plate containing cell culture medium. The measurement of the absorbance of the formazan was performed at 490 nm.

### Glucose uptake

BV2 cells were seeded at a density of 2 × 10^5^ in a 24-well plate and treated with 10 ng/mL LPS. After 3 h later, different concentrations of 2-NBDG (2-(N-(7-Nitrobenz-2-oxa-1,3-diazol-4-yl)Amino))-2-Deoxyglucose prepared in HBSS with 1% FBS media (Invitrogen) were added for 20 min. Cells were washed twice and fixed with 1% PFA. Intensity of fluorescent glucose analog data collected NovoCyte Quanteon flow cytometer (Agiliant), acquire data using NovoExpress software. Median intensity was reported and analyzed.

### Real-time RT PCR

The brain tissues were homogenized using rotor-stator homogenizer isolation. Total RNAs were extracted using the AurumTM Total RNA Mini Kit (BioRad) following the manufacturer’s instructions. The RNAs were quantified using the NanoDrop and their integrities were further confirmed by locating the 18S and 28S bands on a 2% agarose gel. One microgram of total RNA was isolated from cells in culture using the AurumTM Total RNA Mini Kit (BioRad), treated with DNaseI, and reverse transcribed using Superscript II Rnase H-reverse transcriptase (Invitrogen). QPCR was performed using SYBR Green in a 384-well format using a QuantStudio^™^ 6 Flex (Thermo Fisher Scientific). Oligonucleotide primers for QPCR were obtained from Integrated DNA Technologies (Coralville). Primer sequences for GLUT1 and HK2 were validated and used for gene amplification. Levels of mRNA expression were normalized to those of the mouse house-keeping gene, β-actin. Values represent the mean value of triplicate samples +/− SEM. Data are representative of at least two independent experiments. The relative quantification value from each gene was compared to the gene expression of the control BV2 vehicle group.

### α-Syn uptake assay

Human α-syn proteins (rPeptide, Bogart, GA, United States) were assembled into aggregates by incubating at 37°C at concentrations of 1 mg/mL with continuous shaking at 1100 rpm for 7 days. BV2 cells were incubated with 5 μg/mL of α-syn as indicated. Cells were washed with PBS and were lysed in buffer containing 1% Triton X-100 and 1 × protease inhibitor mix (Sigma) for 10 min on ice. Quantitative analysis of the internalized total α-syn aggregates were analyzed by western blot analysis.

### Western blot analysis

Cells were lysed in a buffer containing 1% Triton X-100 and 1 × protease inhibitor mix (Sigma) for 10 min on ice. Lysates were centrifuged at 16,000 g for 5 min at 4°*C. triton* X-100 supernatant was then transferred to a new tube and mixed with 4 × Laemmli sample buffer. The remaining pellet was then washed with ice cold PBS and centrifuged at 16,000 g for 5 min at 4°C. The supernatant was then removed, and the remaining Triton X-100 insoluble pellet was resuspended in 1 × Laemmli sample buffer. Triton X-100 insoluble samples were then sonicated using a high intensity ultrasonic water bath (50% power, 5 s pulses for 1 min) at 4°C prior to being loaded on pre-cast 4–20% SDS electrophoresis gels (Bio-Rad), transferred onto PDVF membranes (Millipore), and probed with α-Syn and β-actin (Santa Cruz biotechnology) and the appropriate HRP-conjugated secondary antibody (1:2000; Jackson ImmunoResearch Lab).

### Statistical analysis

Data were analyzed using Graph Pad Prism (Version 10; GraphPad Software, Inc.; San Diego, CA, United States) and expressed as means ± S.E.M. One-way or two-way analysis of variance (ANOVA) with Tukey HSD (Honestly Significant Difference) or Bonferroni post-hoc tests were used to evaluate mean differences between groups. Significance given at *p* < 0.05 for all figures.

## Results

### Glucose significantly enhances LPS-induced TNF production in RGS10 KD BV2 cells

**Figure 1:**
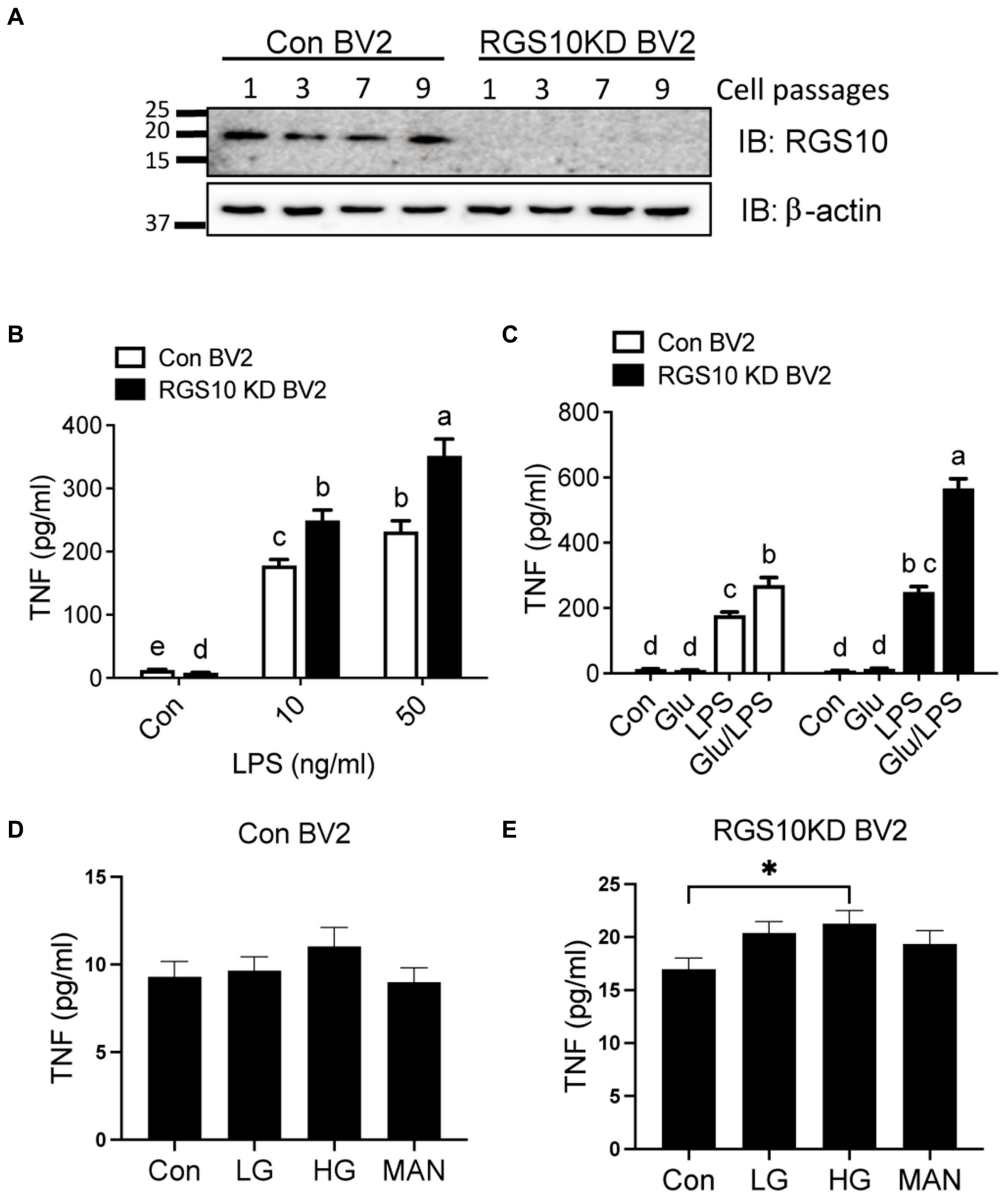
Glucose significantly enhances LPS-induced TNF production in RGS10 KD BV2 cells. **(A)** Western blot data demonstrates a consistent and stable knockdown of RGS10 protein expression across numbers of cell passages in RGS10KD BV2 compared to control BV2 cells. **(B)** Control BV2 and RGS10 KD BV2 cells were treated with vehicle, 10 or 50 ng/mL of LPS for 24 h. The supernatant was collected and TNF levels were measured by ELISA. **(C)** Control BV2 and RGS10 KD BV2 cells were treated with vehicle, glucose (17.5 mM), LPS (10 ng/mL) or a combination of glucose and LPS for 24 h. The supernatant was collected and TNF levels were measured by ELISA. Statistically analysis was conducted using 2-way ANOVA with Bonferroni’s *post hoc* for multiple comparisons, where different letters indicate significant differences among means. **(D,E)** Control BV2 **(D)** and RGS10KD BV2 **(E)** cells were treated with LG (17.5 mM of glucose), HG (35 mM of glucose) or Mannose (35 mM) for 24 h. The supernatant was collected and TNF levels were measured by ELISA. *Denotes significant differences to control group at *p* < 0.05, One-way ANOVA. Values shown in **(B–E)** represent group means (*n* = 6) +/− S.E.M from one experiment representative of three independent experiments.

RGS10 plays a key role in microglia activation (Lee et al., 2008, 2011) and RGS10 knockout mice fed with high-fat diet display glucose intolerance and insulin resistance (Fang et al., 2019). Moreover, the critical role of microglia has been implicated in modulating hypothalamic control of energy homeostasis in high fat diet mice (Valdearcos et al., 2017) and high glucose exacerbates toll-like receptor (TLR) 4-mediated microglia activation (Zhang et al., 2015). Therefore, we aimed to investigate if RGS10 in microglia regulates the inflammatory responses mediated by high-glucose. Here, we utilized a BV2 murine microglia cell line, which was kindly gifted from Dr. Hooks at University of Georgia, that are stably knockdown RGS10 (RGS10 KD BV2) (Alqinyah et al., 2018; Wendimu et al., 2021). The protein expressions of RGS10 in BV2 and RGS10 KD BV2 cells were validated in different passages at 3, 5, 7, and 9 by western blot analysis (Figure 1A). To further validate if RGS10 KD BV2 cells are hyper-activated by LPS, as we have previously shown in primary microglia and transient knockdown in BV2 cells (Lee et al., 2008, 2011), TNF concentrations from supernatants were measured in BV2 control and RGS10 KD BV2 cells treated with LPS. Our data confirmed that RGS10 KD BV2 cells produce significantly higher levels of TNF upon LPS treatments implicating that RGS10 ameliorates TNF production in BV2 cells (Figure 1B). Subsequently, we aimed to investigate whether glucose potentiates LPS-induced TNF production in control BV2 cells and whether glucose further enhances inflammatory response in RGS10 KD BV2 cells. Both control BV2 and RGS10 KD BV2 cells were treated with vehicle, glucose (17.5 mM), LPS (10 ng/mL) or a combination of glucose and LPS for 24 h and TNF levels in supernatants were measured. Our findings revealed that glucose exacerbated TNF production in LPS-treated BV2 cells, indicating a synergistic effect in microglia activation (Figure 1C). Furthermore, RGS10 KD BV2 cells exhibited significantly elevated TNF production compared to control BV2 (Figure 1C), suggesting a regulatory role of RGS10 in glucose metabolism associated with microglia activation. We also investigated whether elevated glucose levels alone could induce microglia activation. BV2 cells were treated with two different glucose concentrations, 17.5 mM and 35 mM, for 24 h, and TNF production were assessed. While high glucose did not induce TNF production in control BV2 cells (Figure 1D), it significantly increased TNF production with high glucose (35 mM)-treated RGS10 KD BV2 cells (*p* < 0.05) (Figure 1E). Mannitol (17.5 mM) was employed as an osmotic control, and no changes in TNF levels were observed in either control or RGS10 KD BV2 cells.

### Glucose uptake and GLUT1 mRNA level are significantly increased upon LPS treatment in RGS10 KD BV2 cells

**Figure 2:**
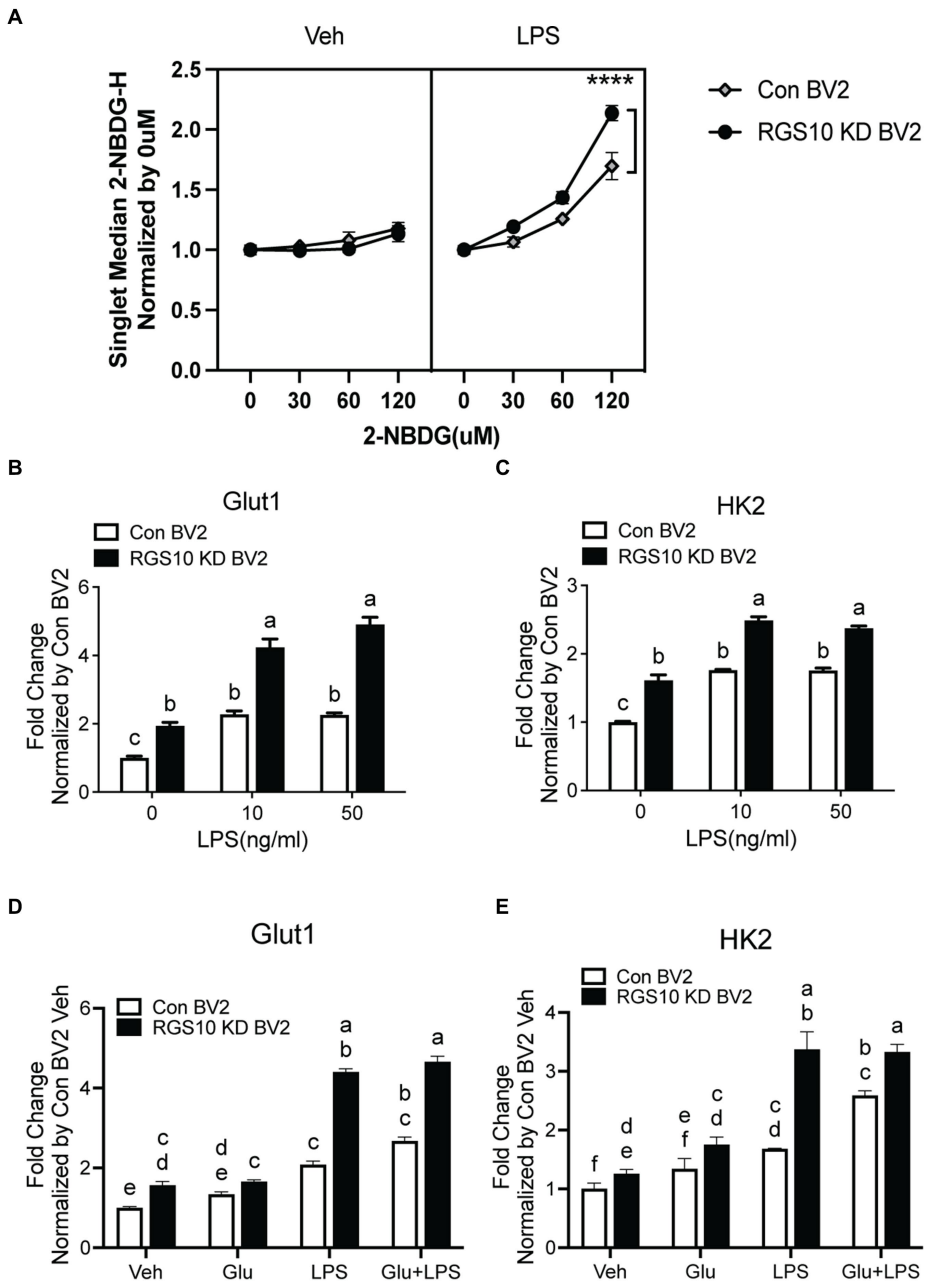
Glucose uptake and GLUT1 mRNA level are significantly increased upon LPS treatment in RGS10 KD BV2 cells. **(A)** Control BV2 and RGS10 KD BV2 cells were treated with LPS (10 ng/mL) for 3 h. Various concentrations of 2-NBDG (0, 30, 60, or 120 μM) were added to the cells for 20 min and 2-NBDG levels were measured by flow cytometry analysis. **** denotes significant differences to control group at *p* < 0.0001, One-way ANOVA. **(B)** Cells were treated with LPS (0, 10, or 50 ng/mL) for 3 h and the mRNA levels of GLUT1 were determined by real-time RT-PCR. **(C)** Cells were treated with LPS (0, 10, or 50 ng/mL) for 3 h and the mRNA levels of HK2 were determined by real time qPCR. **(D)** Cells were treated with glucose (17.5 mM), LPS (10 ng/mL) or the combination of LPS (10 ng/mL) and glucose (17.5 mM) for 3 h and the mRNA levels of GLUT1 were determined by real time qPCR. **(E)** Cells were treated with glucose (17.5 mM), LPS or the combination of LPS (10 ng/mL) and glucose (17.5 mM) for 3 h and the mRNA levels of GLUT1 were determined by real time qPCR. Values shown in **(B–D)** represent group means (*n* = 6) +/− S.E.M from one experiment representative of three independent experiments. Statistically analysis was conducted using 2-way ANOVA with Bonferroni’s *post hoc* for multiple comparisons, where different letters indicate significant differences among means.

During inflammatory responses, microglia switch from using mitochondrial oxidative phosphorylation (OXPHOS) in resting states to aerobic glycolysis (Orihuela et al., 2016; Cheng et al., 2021). To investigate whether RGS10 knockdown in microglia facilitates glucose uptake upon inflammatory stimulation, we assessed glucose uptake in RGS10 KD BV2 cells upon various concentrations of LPS treatments. Both control BV2 and RGS10 KD BV2 cells were treated with LPS (10 ng/mL) for 3 h. To monitor glucose uptake in live cells, we employed a fluorescent glucose analog, 2[N-(7-nitrobenz-2-oxa-1,2-diaxol-4-yl)amino]-2-deoxyglucose (2-NBDG). Cells were treated with various concentrations of 2-NBDG (0, 30, 60, or 120 μM) for 20 min, and 2-NBDG levels within the cells were quantified by flow cytometry. Glucose uptake was increased in control BV2 cells upon LPS treatment, and significantly enhanced glucose uptake was observed in RGS10 KD BV2 cells compared to control BV2 cells (Figure 2A) (*p* < 0.0001). In microglia, GLUT1 is responsible for facilitating glucose uptake during inflammatory conditions, and silencing GLUT-1 and HK2 abolishes LPS-induced microglial cell activation (Guan and Han, 1999). Hence, we examined the mRNA levels of GLUT1 and HK2 in RGS10 KD microglia following LPS. Both control BV2 and RGS10 KD BV2 cells were subjected to LPS (0, 10 or 50 ng/mL) for 3 h, and the mRNA levels of GLUT1 and HK2 were assessed using real-time RT-PCR. Our results demonstrated a significant increase in the mRNA levels of GLUT1 and HK2 in control BV2 cells and RGS10 KD BV2 cells upon LPS treatments (Figures 2B,C). We also noted that the baseline mRNA levels of GLUT1 and HK2 are higher in RGS10 KD BV2 cells compared to control BV2 cells (Figures 2B,C).

To investigate the impact of glucose on the mRNA levels of GLUT1 and HK2, control BV2 and RGS10 KD BV2 cells were treated with glucose, LPS or a combination of LPS (10 ng/mL) and glucose (17.5 mM) for 3 h and the mRNA levels of GLUT1 and HK2 were analyzed using real-time RT-PCR. Our results demonstrated that both GLUT1 and HK2 levels were significantly higher upon glucose, LPS and a combination of LPS and glucose treatments in RGS10 KD BV2 cells compared to control BV2 cells (Figures 2D,E). However, glucose did not augment the LPS-induced effect on mRNA levels of GLUT1 and HK2 in either RGS10 KD BV2 cells control BV2 cells (Figures 2D,E).

### ROS was significantly increased and led to TNF production in RGS10 KD BV2 cells treated with glucose and LPS

**Figure 3:**
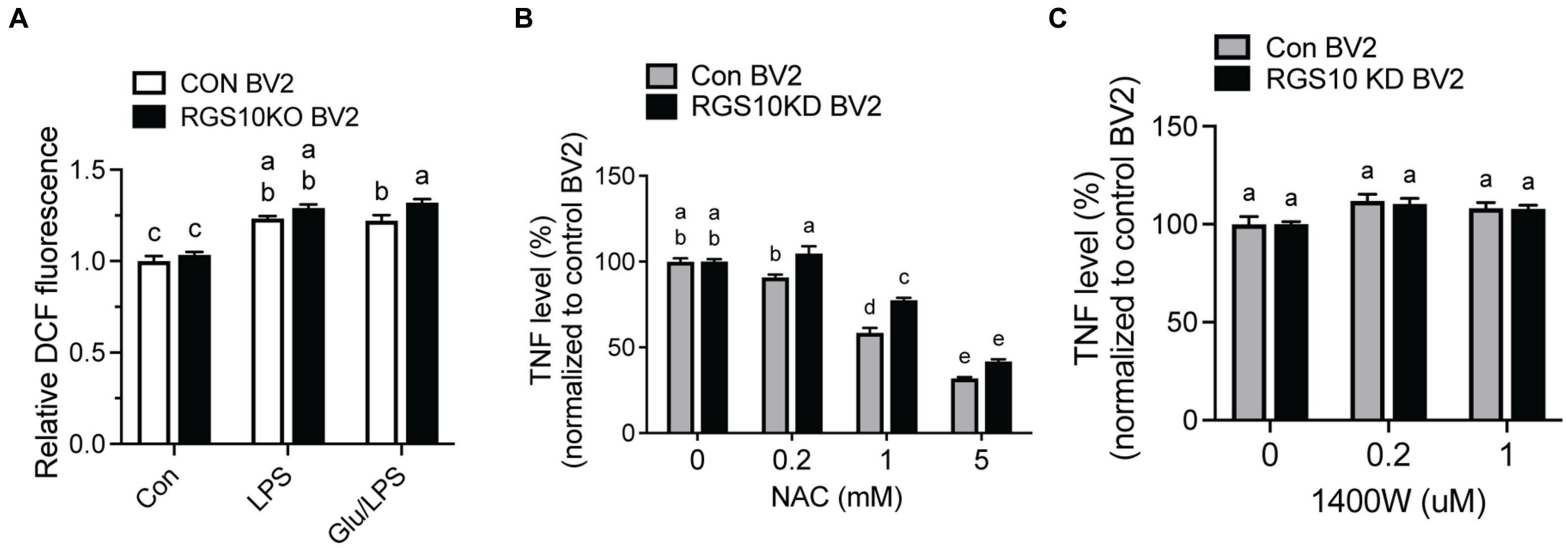
ROS was significantly increased and led to TNF production in RGS10 KD BV2 cells upon glucose and LPS treatments. **(A)** Control BV2 and RGS10 KD BV2 cells were treated with 17.5 mM glucose and ROS levels were assessed by measuring fluorescent compound 2′,7’dichlorofluorescin (DCF). **(B)** Control BV2 and RGS10 KD BV2 cells were pretreated with vehicle or NAC (0, 0.2, 1 or 5 mM) for 30 min and then treated with the combination of glucose (17.5 mM) and LPS (10 ng/mL) for 24 h. The supernatant was collected and TNF levels were measured by ELISA and values were normalized to control BV2 treated with the combination of glucose (17.5 mM) and LPS (10 ng/mL). **(C)** Control BV2 and RGS10 KD BV2 cells were pretreated with 1,400 W (0, 0.2, or 1 μM) for 30 min and then treated with 17.5 mM glucose and or 10 ng/mL LPS for 24 h in 37°C. The supernatant was collected and TNF levels were measured by ELISA and values were normalized to control BV2 treated with the combination of glucose (17.5 mM) and LPS (10 ng/mL). Values shown in **(A–C)** represent group means (*n* = 6) +/− S.E.M from one experiment representative of three independent experiments. Statistically analysis was conducted using 2-way ANOVA with Bonferroni’s *post hoc* for multiple comparisons, where different letters indicate significant differences among means.

When microglia are exposed to high glucose, microglia produce ROSs along with inflammatory cytokines (Yang et al., 2020; Kongtawelert et al., 2022). To understand the mechanism of RGS10, we examined whether RGS10 modulates ROS production. RGS10 KD BV2 or control BV2 cells were treated with vehicle, glucose (17.5 mM), LPS (10 ng/mL) or the combination of glucose (17.5 mM) and LPS (10 ng/mL) for 24 h. ROS levels were assessed by measuring fluorescent compound 2′,7’dichlorofluorescin (DCF). Our results showed a significant increase of ROS production in RGS10 KD BV2 cells in response to the combinations of glucose and LPS compared to control BV2 cells (Figure 3A). Then, we further examined if the ROS inhibitor, N-Acetylcysteine (NAC), differentially affects microglia activation in control BV2 and RGS10 KD BV2 cells by measuring TNF production. Both RGS10 KD BV2 or control BV2 cells were pretreated with vehicle or NAC (0, 0.2, 1 or 5 mM) for 30 min and then treated with the combination of glucose (17.5 mM) and LPS (10 ng/mL) for 24 h. TNF levels in the supernatants were measured by ELISA. Our data showed that TNF levels were decreased in a dose-dependent manner upon NAC treatment in both control BV2 and RGS10 KD BV2 cells (Figure 3B). Additionally, the antioxidant effect of NAC was significantly diminished in RGS10 KD BV2 cells compared to control BV2 cells (Figure 3B), suggesting that RGS10 negatively regulates microglial activation not only by reducing the NF-kB signaling pathway (Fang et al., 2019) but also by attenuating ROS production. To test whether nitric oxide (NO) is involved in the process, we tested the effect of NO synthase inhibitor, 1,400 W dihydrochloride. To do so, RGS10 KD BV2 or control BV2 cells were pretreated with 1,400 W dihydrochloride (0, 0.2, or 1 μM) for 30 min, followed by treatment with the combination of glucose (17.5 mM) and LPS (10 ng/mL) for 24 h. Our results showed that NO synthesis inhibitor did not alter TNF production in either RGS10 KD BV2 or control BV2 cells, suggesting that NO is not directly involve in TNF production induced by the combinations of glucose and LPS (Figure 3C).

### Glucose significantly enhances α-Syn-induced TNF production in RGS10 KD BV2 cells

**Figure 4:**
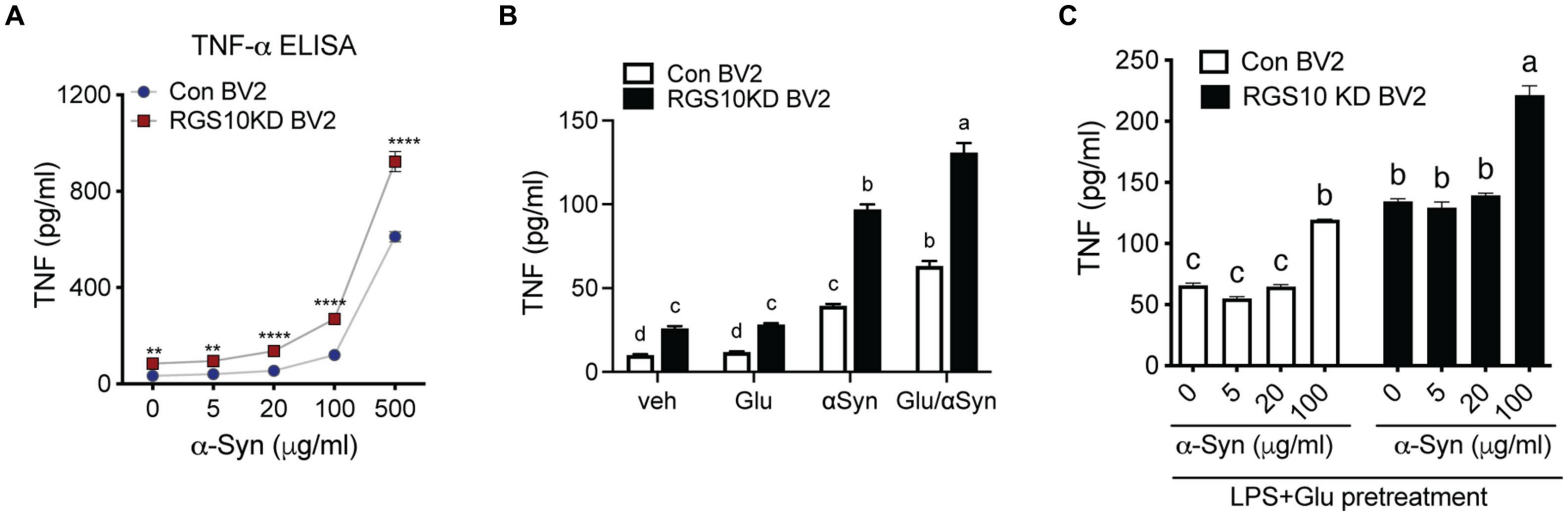
Glucose significantly enhances α-syn-induced TNF production in RGS10 KD BV2 cells. **(A)** Control BV2 and RGS10 KD BV2 cells were treated with α-syn (1, 10, 40, or 160 μg/mL) for 24 h and conditioned medium was collected. The supernatant was collected and TNF levels were measured by ELISA. *p < 0.05, **p < 0.01, ***p < 0.001, and ****p < 0.0001 compared between the groups, two-way ANOVA with Bonferroni *post hoc* for multiple comparisons. **(B)** Control BV2 and RGS10 KD BV2 cells were treated with PBS, α-syn (40 μg/mL) or α-syn (40 μg/mL)/glucose (17.5 mM) for 24 h. TNF productions in conditioned medium were measured by ELISA. Statistically analysis was conducted using 2-way ANOVA with Bonferroni’s *post hoc* for multiple comparisons, where different letters indicate significant differences among means. **(C)** RGS10 KD BV2 cells or control BV2 cells were treated with α-syn (40 μg/mL) with or without the combination of glucose (17.5 mM) and LPS (10 ng/mL) for 24 h. The supernatant was collected and TNF levels were measured by ELISA. Values shown represent group means (*n* = 6) +/− S.E.M from one experiment representative of three independent experiments. *p < 0.05 compared within the group. Statistically analysis was conducted using 2-way ANOVA with Bonferroni’s *post hoc* for multiple comparisons, where different letters indicate significant differences among means.

Extracellular alpha-synuclein (α-syn) has been detected in the plasma and CSF of PD and MSA patients and has been shown to modulate immune responses. In the CNS, neurons and glial cells can take up extracellular α-syn; however, α-syn internalization will result in inclusions in neurons and pro-inflammatory response in glial cells (Desplats et al., 2009; Lee et al., 2010). Oligomeric α-syn produced by neurons are phagocytosed and activate neighboring microglia (Kim et al., 2013). Microglia and macrophages are able to uptake and degrade extracellular α-syn (Fellner et al., 2013; Kim et al., 2013). The effect of α-syn on immune cells is not simply mediated by phagocytosis of non-specific protein debris but by specific receptors and their downstream pathways. To investigate the involvement of RGS10 in α-syn-mediated microglia activation, we assessed whether α-syn enhances microglia activation. RGS10 KD BV2 cells or control BV2 cells were treated with different concentrations of α-syn (1, 10, 40, or 160 μg/mL) for 24 h, and supernatants were collected to measure TNF production. Our results revealed that TNF levels were increased in a dose-dependent manner upon α-syn treatments in both control BV2 and RGS10 KD BV2 cells, with significantly higher levels observed in RGS10 KD BV2 cells compared to control BV2 cells (Figure 4A).

Subsequently, we examined whether glucose enhances microglia activation induced by α-syn. To test this, RGS10 KD BV2 or control BV2 cells were treated with PBS, glucose (17.5 mM), α-syn (40 μg/mL) or the combination of α-syn (40 μg/mL) and glucose (17.5 mM) for 24 h, and supernatants were collected to measure TNF production. Our data shows that glucose significantly enhances α-syn-induced TNF production in both control BV2 and RGS10 KD BV2 cells, moreover, the levels of TNF production were significantly higher in RGS10 KD BV2 cells (Figure 4B).

Next, we tested whether LPS and glucose pretreatment enhances α-syn-induced TNF production in RGS10 KD BV2 cells. RGS10 KD BV2 cells or control BV2 cells were pre-treated with glucose (17.5 mM) and LPS (10 ng/mL) for 24 h, followed by exposure to different concentrations of α-syn (1, 5, 20, or 100 μg/mL) for an additional 24 h (Figure 4C). Our data demonstrated that the pretreatment of glucose and LPS led to significantly higher α-syn-induced TNF production in RGS10 KD BV2 cells compared to control BV2 cells.

### RGS10 KD BV2 cells exhibit impaired internalization of extracellular α-Syn, and the presence of glucose/LPS significantly exacerbates this effect

**Figure 5:**
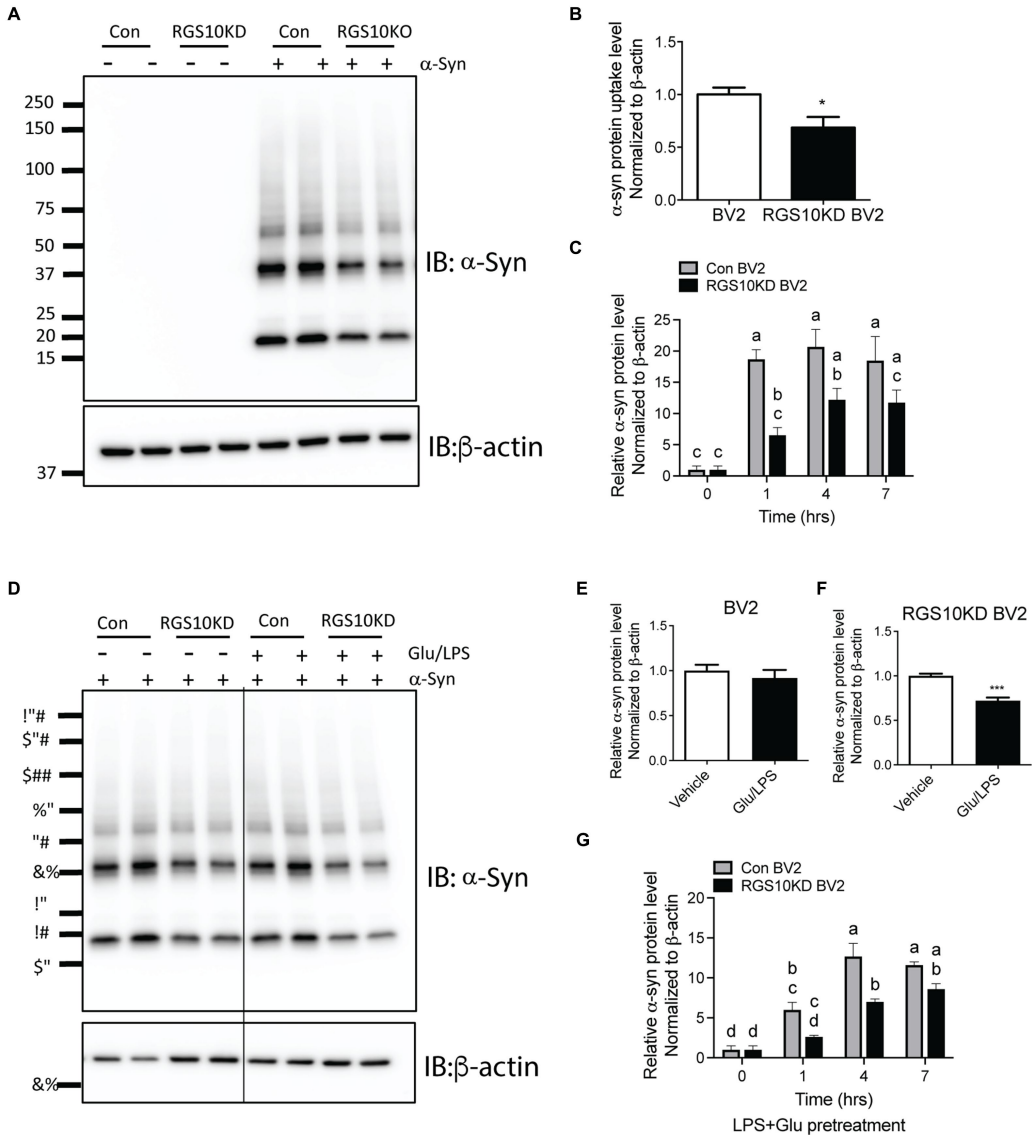
RGS10 KD BV2 cells exhibit impaired internalization of extracellular α-syn, and the presence of glucose/LPS significantly exacerbates this effect. **(A,B)** Control BV2 and RGS10 KD BV2 cells were treated with α-syn (5 μg/mL) for 1 h. Cells were washed three times with PBS to eliminate membrane-bound α-syn and internalized α-syn were analyzed by western blot analysis for α-syn **(A)** and quantitation analyses **(B)**. **(C)** Control BV2 and RGS10 KD BV2 cells were incubated with α-syn (5 μg/mL) for 1, 4 and 7 h. Time-dependent internalized α-syn were analyzed by immunoblot analysis. **(D–F)** Control BV2 and RGS10 KD BV2 cells were treated were treated with the combination of glucose (17.5 mM) and LPS (10 ng/mL) for 24 h, then α-syn (5 μg/mL) were treated for 1 h. Internalized α-syn were analyzed by immunoblot analysis **(D)** and quantitation analyses for control BV2 **(E)** and RGS10 KD BV2 cells **(F)**. **(G)** RGS10 KD BV2 cells or control BV2 cells were treated were treated with the combination of glucose (17.5 mM) and LPS (10 ng/mL) for 24 h, then α-syn (5 μg/mL) were treated for 1 h, 4 h, and 7 h. Time-dependent internalized α-syn were analyzed by immunoblot analysis. Western blot data shown represent the representational data from three-independent experiments. Statistically analysis was conducted using 2-way ANOVA with Bonferroni’s *post hoc* for multiple comparisons, where different letters indicate significant differences among means.

Microglia efficiently scavenge extracellular monomeric and aggregated forms of α-syn (Fellner et al., 2013; Kim et al., 2013). Our data indicates that the absence of RGS10 in BV2 cells results in an increase in proinflammatory cytokines and ROS production upon LPS treatments, and these phenotypes are further exacerbated under high glucose conditions. To determine whether high glucose or RGS10 deficiency affects clearance of synuclein by microglia, we measured internalization of a-syn species *in vitro*, including various sizes (monomer, oligomers, and higher molecular weight of fibrils) as described previously (Earls et al., 2019). RGS10 KD BV2 cells or control BV2 cells were treated with α-syn (5 μg/mL) for 1 h. Cells were washed three times with PBS to eliminate membrane bound α-syn and internalized α-syn were analyzed by immunoblot analysis. Our results showed that BV2 cells efficiently internalized various sizes of α-syn aggregates (Figure 5A). RGS10 KD BV2 cells demonstrated significantly impaired internalization of α-syn compared to control BV2 cells (Figure 5B). Next, to monitor the internalization of α-syn over time, cells were incubated with α-syn (5 μg/mL) for 1, 4 and 7 h. Our data showed that both control BV2 and RGS10 KD BV2 cells were able to internalize α-syn, however, RGS10 KD BV2 cells showed significantly impaired internalization of α-syn compared to control BV2 cells over time (Figure 5C).

**Figure 6:**
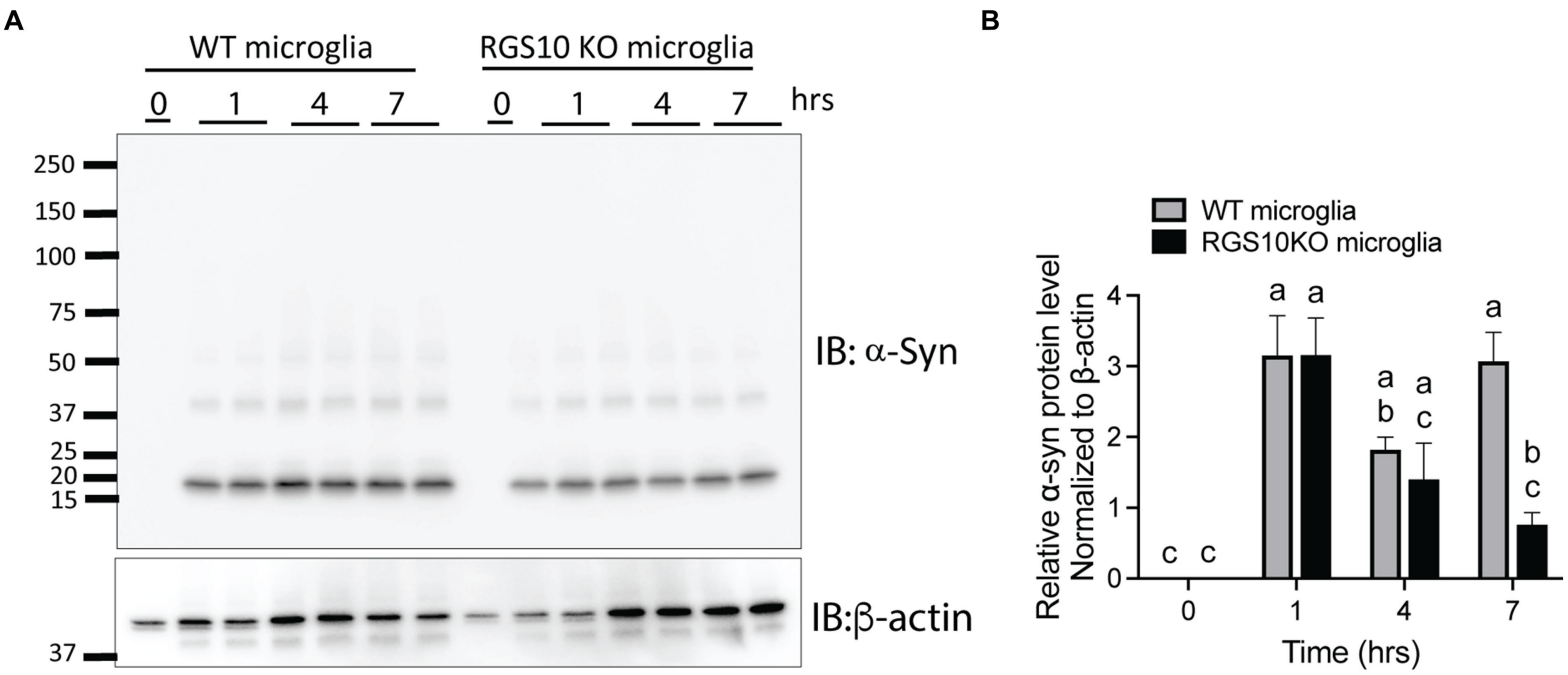
RGS10 knockout primary microglia exhibit impaired internalization of extracellular α-syn. **(A)** Primary microglia from WT and RGS10 ko mice were treated with α-syn (5 μg/mL) for 1, 4, and 7 h. After washing cells three times with PBS to eliminate membrane-bound α-syn, internalized α-syn was analyzed by immunoblot analysis. **(B)** Quantitative data of internalized α-syn were analyzed by immunoblot analysis. Western blot data shown represent the representational data from two-independent experiments. Statistically analysis was conducted using 2-way ANOVA with Bonferroni’s *post hoc* for multiple comparisons, where different letters indicate significant differences among means.

Then, we tested whether the combination of glucose and LPS changes the internalization of α-syn aggregates. RGS10 KD BV2 cells or control BV2 cells were treated with the combination of glucose (17.5 mM) and LPS (10 ng/mL) for 24 h, then α-syn (5 μg/mL) were treated for 1 h. Our data showed that the combination of glucose (17.5 mM) and LPS (10 ng/mL) significantly impairs internalization of α-syn aggregates in RGS10 KD BV2 cells but not in control BV2 cells (Figures 5D–F). To measure the internalization of α-syn over time, RGS10 KD BV2 cells or control BV2 cells were treated were treated with the combination of glucose (17.5 mM) and LPS (10 ng/mL) for 24 h, then α-syn (5 μg/mL) were treated for 1 h, 4 h, and 7 h. The combination of glucose and LPS treatment resulted in further impairment of α-syn internalization at 1 h and 4 h (Figure 5G) implicating diminished overall clearance of α-syn. To validate these findings in primary microglia, primary microglia from WT mice and RGS10 KO mice were pretreated with a combination of glucose (17.5 mM) and LPS (10 ng/mL) for 24 h, followed by α-syn (5 μg/mL) treatments for 1 h, 4 h, and 7 h. The combination of glucose and LPS similarly led to a diminished internatlization of α-syn at 1 h and 7 h (Figures 6A,B) indicating a compromised α-syn clearance in primary microglia lacking RGS10.

## Discussion

Neurodegenerative diseases are accompanied by a slew of dysregulated cellular and molecular mechanisms including chronic inflammation, protein aggregation, and metabolic dysfunction. These mechanisms are not present in isolation but highly interconnected, synergistically advancing disease processes (Wilson et al., 2023). Here we examine the ability for RGS10 to maintain microglial homeostasis under multiple disease associated conditions. From this study we demonstrate that RGS10 mediates the inflammatory response, ROS production, and internalization of protein aggregates in microglia under inflammatory and hyperglycemic conditions.

### RGS10 mitigates the microglia inflammatory response under a host of disease associated states

Recent studies have increasingly implicated impaired glucose metabolism in neurodegenerative diseases, with a particular emphasis on the potential role of hyperglycemia to induce microglial activation and neuroinflammation (Sergi et al., 2019; Hernandez-Rodriguez et al., 2022; Dai et al., 2023). Moreover, protein aggreagates such as α-syn have been shown to induce microglial activation as well (Lv et al., 2022). Previous studies have demonstrated that RGS10 maintains microglial homeostasis by negatively regulating the NF-κB pathway (Lee et al., 2011). Interestingly, we have recently shown that RGS10 helps prevent metabolic dysfunction in mice fed a high fat diet, indicating a role for RGS10 in glucose metabolism (Fang et al., 2019). Here we show that inflammatory stimuli, hyperglycemic conditions, and α-syn aggregates synergistically increase TNF production in BV2 cells that is mitigated by RGS10.

### RGS10 mitigates glycolysis and the production of ROS in hyperglycemic and inflammatory conditions in microglia

Recent research has indicated that, during inflammatory responses, microglia transition from using mitochondrial oxidative phosphorylation (OXPHOS) in resting states to relying on anaerobic glycolysis when microglia are stimulated with LPS (Orihuela et al., 2016). In the mammalian brain, glucose serves as the primary energy source (Mergenthaler et al., 2013), and neurons predominantly utilize glucose through the glucose transporter 3 (GLUT3) (Mergenthaler et al., 2013). However, during CNS inflammation, activated microglia exhibit high levels of glycolysis, which not only support the generation of harmful inflammatory molecules but also consume substantial amounts of glucose that are vital for neurons. Understanding the metabolic pathways of activated microglia will be crucial for effectively controlling neuroinflammation and improving the management of neurodegenerative conditions. Glucose uptake in microglia is facilitated predominately by GLUT1, particularly under inflammatory conditions (Wang et al., 2019). Therefore, targeting GLUT1 could be an effective approach to control neuroinflammation (Wang et al., 2019). In our study, we observed that RGS10-deficient BV2 cells significantly increased florescent glucose uptake upon inflammatory stimulation as well as GLUT1 and HK2 mRNA expression under both inflammatory and hyperglycemic conditions. Given that exacerbated glycolysis and hyperglycemic conditions can stimulate ROS production, we conducted a series of experiments to determine whether RGS10 influenced ROS production and depletion. Our results demonstrate that RGS10-deficient BV2 cells produce higher levels of ROS, and the antioxidant effect on TNF production was significantly reduced in these cells. These results demonstrate that RGS10 plays a role in mitigating glycolysis and ROS production in microglia under hyperglycemic and inflammatory conditions.

### RGS10 helps facilitate the internalization of α-Syn aggregates even under hyperglycemic and inflammatory conditions

As the major phagocytes of the CNS, microglia play a critical role in clearing any aberrant protein species associated with neurodegenerative diseases (Hickman et al., 2018). Both systemic inflammation and hyperglycemic conditions have been found to reduce the clearance capacity of microglia in preclinical AD models and mixed glial cultures, respectively, (Tejera et al., 2019; Xie et al., 2021; Huang et al., 2022). Furthermore, both systemic inflammation and hyperglycemia have shown to exacerbate α-syn aggregation mouse models (Kim et al., 2022; Lv et al., 2022). RGS10 has been shown to be important for full phagocytotic capacity of M2 macrophages (Lee et al., 2013) but there is a current lack in understanding whether lysosomal function and protein clearance is compromised with RGS10 deficiency. Here we show for the first time, that RGS10 supports the clearance and uptake of α-syn aggregates in BV2 cells even under hyperglycemic and inflammatory conditions. As this study was primarily completed with the use of a murine microglial cell line, BV2 cells, and ex vivo primary microglia, further experiments conducted *in vivo* and in translationally relevant human models should be followed to ensure generalizability of our results outside of in vitro conditions. Moreover, our study examines only one cytokine, TNF, to assess the inflammatory profile of microglia which reduces our ability to interpret the full inflammatory response.

### Mechanisms, inferences, and implications

RGS10 is found all throughout the cell and was recently identified to have multiple non-canonical protein binding partners, indicating that RGS10 may be capable of maintaining microglial homeostasis in response to a wide range of stimuli (Alqinyah et al., 2018). RGS10 modulates TNF production by restraining the NF-kB signaling pathway in microglia, despite lacking direct interaction with NF-kB components (Lee et al., 2011). Wendimu et al. demonstrated that RGS10 interacts with stromal interaction molecule 2 (STIM2), diminishing cytoplasmic calcium influx and thus reducing microglial activation (Wendimu et al., 2021). RGS10 also interacts with phosphodiesterase 4A (PDE4A), which hydrolyzes cAMP to AMP (Wendimu et al., 2021). PKA signaling exerts anti-inflammatory effects via the enhancement of cAMP-response element-binding protein (CREB) and nuclear factor erythroid 2-related factor 2 (Nrf2) activities, inhibiting NF-kB signaling pathway, thereby mitigating inflammation and cellular oxidative stress (Tavares et al., 2020). Moreover, RGS10 enhances PKA-dependent CREB activation in neurons suggesting RGS10’s involvement in modulating the upstream signaling of the PKA pathway (Lee et al., 2012). While further investigation is warranted to ascertain whether RGS10 directly governs glucose uptake, it is established that GLUT1-mediated glucose uptake augments microglial activation (Wang et al., 2019). Notably, our findings unveiled elevated levels of GLUT1 and HK2 mRNA, as well as increased glucose uptake in RGS10-deficient microglia following LPS stimulation, hinting at potential transcriptional regulation of these genes by RGS10.

Given previous studies demonstrating that RGS10 is a crucial homeostatic protein in microglia (Butovsky et al., 2014) but its decreased expression in microglia with age as well as in mouse models of neurodegenerative diseases such as MS, AD, and ALS (Kannarkat et al., 2015; Krasemann et al., 2017), it is reasonable to anticipate that increasing RGS10 levels could hold therapeutic potential. This becomes particularly evident when considering the functional changes observed in microglia lacking RGS10. Previous research has revealed a reduction in Fc-δ receptor mediated phagocytosis of RGS10-null primary microglia (Lee et al., 2011), alongside a significant increase in cytokine production upon stimulation in these cells (Lee et al., 2008). Regarding therapeutic delivery of RGS10, lentiviral vectors for RGS10 have been demonstrated to mitigate the development of microgliosis and prevent dopaminergic cell loss in the substantia nigra in a parkinsonian rat model (Lee et al., 2011). A study from the Hooks lab has demonstrated that sphingosine-1-phosphate (S1P) can increase the transcription of RGS10, possibly by inhibiting the histone deacetylase activity. Taken together, these studies suggest potential avenues for therapeutically modulating RGS10 in patient populations, with the aim of targeting neuroinflammation by modulating energetic shifts and classical inflammatory pathways in microglia.

Furthermore, we have observed that RGS10’s regulation of microglial activation is distinct within its family of RGS proteins. Specifically, RGS10 is the most abundantly expressed RGS transcript in microglia and myeloid cells (Lee et al., 2013). Additionally, our finding indicates that the paracrine components of conditioned media from BV2 cells exposed to LPS, with a transient knock down of RGS10 but not RGS4, can diminish the viability of dopaminergic cell lines (Lee et al., 2011). These observations underscore the unique role of RGS10 in modulating microglial function and its potential significance in neuroinflammatory processes.

This study contributes to the growing understanding of RGS10’s role in maintaining microglial homeostasis under disease-related conditions. Further studies are needed to fully comprehend the regulatory capacity of RGS10 and its potential impact on diseases associated with chronic neuroinflammation, i.e., neurodegenerative diseases. Given the pathological significance of microglial activation and the expanding role of RGS10 to maintain microglial homeostasis in response to various pathological factors such as chronic inflammation and metabolic dysregulation, RGS10 may indeed serve as a promising therapeutic target.

## Data availability statement

The original contributions presented in the study are included in the article/Supplementary material, further inquiries can be directed to the corresponding author.

## Author contributions

JC: Project administration, Methodology, Formal analysis, Conceptualization, Writing – review & editing, Writing – original draft. JJ: Writing – review & editing, Methodology, Data curation. KM: Writing – review & editing, Methodology. J-KL: Writing – review & editing, Writing – original draft, Supervision, Resources, Investigation, Funding acquisition, Conceptualization.
