# Supplementary figures and images for: RGS10 mitigates high glucose-induced microglial inflammation via the reactive oxidative stress pathway and enhances synuclein clearance in microglia
Source: Front Cell Neurosci. 2024 May 15;18:1374298. doi: 10.3389/fncel.2024.1374298 (PMC11133718; doi:10.3389/fncel.2024.1374298)

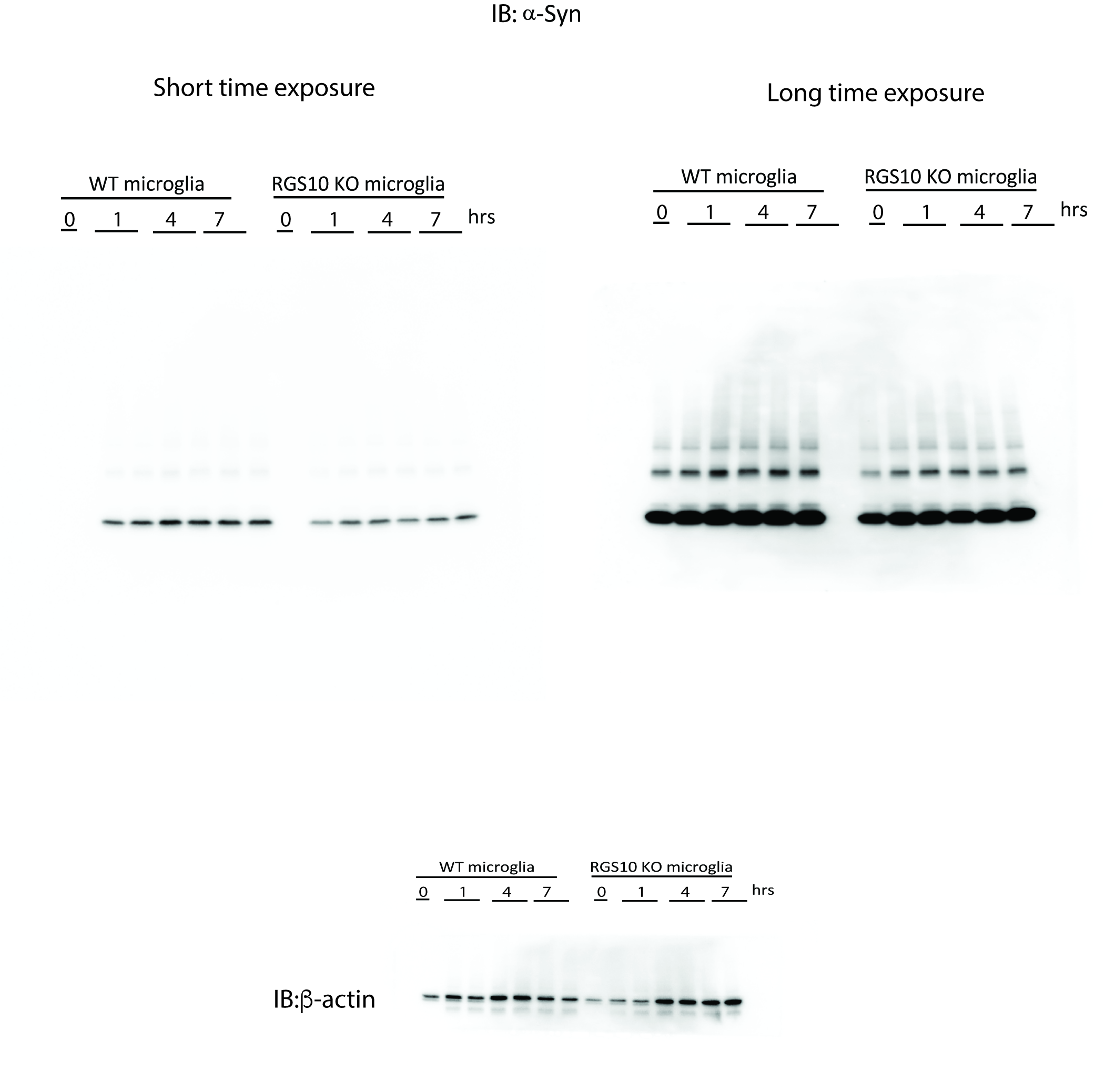

Supplement: Supplementary file 4 [file Image_4.tif]
